# U-Shaped Tube Based Liquid–Solid Triboelectric Nanogenerator for Harvesting Unutilized Compressed Air Energy

**DOI:** 10.3390/mi14112057

**Published:** 2023-11-02

**Authors:** Xuhang Cai, Zhijian Liu, Jingming Dong, Haoji Li, Jiamu Han, Jiaming Huang, Haotian Chen

**Affiliations:** Marine Engineering College, Dalian Maritime University, Dalian 116026, China; dmucxh@dlmu.edu.cn (X.C.); 0741lhj@dlmu.edu.cn (H.L.); han0814@dlmu.edu.cn (J.H.); jiaming_huang@dlmu.edu.cn (J.H.); hotachen@dlmu.edu.cn (H.C.)

**Keywords:** liquid–solid triboelectric nanogenerator, U-shaped tube, unutilized compressed air energy harvesting

## Abstract

Due to a lack of technologies that harvest green and sustainable energy, unutilized compressed air energy during the operation of pneumatic systems is wasted. Liquid–solid triboelectric nano-generators (L-S TENGs) have been widely used as an advanced technology with broad development prospects due to their advantages of a simple structure and long service life. Among them, liquid–solid triboelectric nanogenerators with tube structures have great potential for coupling multiple physical effects and integrating them into a single device. Herein, a U-shaped tube triboelectric nanogenerator composed of fluorinated ethylene propylene (FEP) and copper foil (UFC-TENG) is proposed to directly harvest unutilized compressed air energy. The UFC-TENG can collect unutilized compressed air energy with a stable peak voltage and current of approximately 33 V and 0.25 μA, respectively. When the alternating frequency of the liquid is 0.9 Hz, the unutilized compressed air can drive the UFC-TENG unit with an inner diameter of 12 mm, achieving a maximum output power of 3.93 μW at an external load resistance of 90 MΩ. The UFC-TENG is a novel driving method for L-S TENGs and demonstrates the promising potential of TENGs in the harvesting of unutilized compressed air energy in pneumatic systems.

## 1. Introduction

The triboelectric nanogenerator (TENG), invented by Zhonglin Wang and his coworkers in 2012, can effectively harvest and convert various low-frequency and high-entropy energies into electric energy [1]. The TENG, as a novel energy harvesting technology, has attracted the attention of researchers around the world due to its unique advantages, such as its light weight, cost-effectiveness, and convenient selection of materials [2,3,4,5]. However, the traditional solid–solid triboelectric nanogenerator (S-S TENG) based on the triboelectric effect has been greatly influenced by environmental factors [6], especially ambient temperature and humidity [7,8,9], in terms of output performance and lifespan. Furthermore, the consecutive physical friction or collision of the triboelectric electrode also has an adverse effect on the wear resistance and stability of the TENG [10,11]. 

In this regard, the superiority of the liquid–solid triboelectric nanogenerator (L-S TENG) is greater. Compared with S-S TENGs, L-S TENGs contain liquid material with excellent fluidity, which can effectively increase the contact separation area, thus making the contact electrification at the liquid–solid interface more significant. Hence, L-S TENGs have the potential to be used as energy-harvesting devices [12] that convert various forms of mechanical energy, such as rain droplets [13,14,15], sea waves [16,17,18,19], and human kinetic energy [20,21,22], into electrical energy. Furthermore, they can also be applied in various fields such as flexible wearable technologies, self-powered sensing [23,24,25,26], and electrochemical protection [27]. 

In this work, a simple U-shaped tube triboelectric nanogenerator composed of fluorinated ethylene propylene (FEP) and copper foil (UFC-TENG) is designed to directly harvest energy from unutilized compressed air. The UFC-TENG achieves a high-performance output by using ultrapure water and FEP as positive and negative triboelectric materials, respectively. Compared with traditional L-S TENGs, the U-shaped tube structure of the UFC-TENG exhibits stronger robustness and is less affected by environmental factors, which can greatly improve the superiority of its application. In addition, the UFC-TENG can achieve practical applications such as DC output, capacitor charging, and signal acquisition in the case of low-frequency differential pressure drives when combined with different external circuit designs. Compared with mechanical vibration, the disturbance of differential pressure driving on TENGs is weaker, but its output performance is better. This work indicates that the UFC-TENG we designed provides a potential pathway to harvest low-frequency mechanical energy. Moreover, the multi-functional application of the UFC-TENG shows that TENGs have broad application prospects in pneumatic systems.

## 2. Materials and Methods

### 2.1. Fabrication of the UFC-TENG

Three straight FEP tubes with an outer diameter of 14 cm and a wall thickness of 1 mm and two 90° right angle elbows were used to fabricate the U-shaped tubular TENG with a total length of 70 cm. The straight FEP tubes have various inner diameters (4 mm, 6 mm, 8 mm, 10 mm, 12 mm, 14 mm, and 16 mm) and are adjustable in length (5 cm, 10 cm, 15 cm, 20 cm, 25 cm, 30 cm, 35 cm, 40 cm, 45 cm, and 50 cm). Two copper foil electrodes with a length of 10 cm were pasted at 5 cm and 15 cm from the bottom tube, respectively (50 μm in thickness). The wires were used to connect to the external load, which was powered by the copper foil electrodes. Ultrapure water was poured into the U-shaped tubular TENG so that the liquid level was located at the highest point of the copper foil electrode, which is 5 cm away from the bottom of the tube. The high transparency of FEP tubes makes them suitable for observing the liquid in the tube. It should be noted that all the FEP tubes and elbows used in the experiment were cleaned ultrasonically in ethanol for 10 min.

### 2.2. Characterization and Measurement

The experimental system used in this work is shown in Figure S1. Herein, an air compressor (TBSHIELD, Jinhua, China), a two-position five-way pneumatic control valve (ZDV-08), and a standard cylinder (SC63X50) were used to simulate the operation of the typical pneumatic system. The pressure difference at both ends of the U-shaped tube was generated by the ejector (ACV-25HS), which was driven by the compressed air discharged from the two-position, five-way pneumatic control valve. The alternating frequency of the liquid was adjusted by changing the operating frequency of the cylinder. The output voltage was measured using an oscilloscope (Tektronix, Beaverton, OR, USA, TBS2104B) equipped with a high-attenuation ratio probe (100 MΩ). The output current was measured using a micro-current tester (QH-05 Ver 2.0) and a current amplifier (VK403). An oscilloscope was used to observe this (Tektronix, Beaverton, OR, USA, TBS2104B). A charge amplifier (VK105) was used to measure the transferred charges during the operation of the UFC-TENG, and an oscilloscope (Tektronix, TBS2104B) was used to observe it. The nominal power of the UFC-TENG was verified using a high-precision resistance box (MC-21-B). The contact angle between water and FEP was measured using a contact angle meter (FM40Mk2). During the experiment, the relative humidity was approximately 60%, and the ambient temperature was approximately 23.0 °C. The printed circuit boards were customized based on the designed circuit, and different numbers of LEDs were soldered on them. The potential distribution and flow field were simulated using COMSOL Multiphysics 6.0 software.

## 3. Results and Discussion

### 3.1. Structure Design and Working Principles of the UFC-TENG

The application of pneumatic systems is widespread across various fields of production activities, wherein the gas discharged during operation contains a certain amount of energy [28]. However, due to the low discharge pressure (the relative pressure is approximately 0.1 MPa) and the small flow rate, this part of the gas has not been effectively utilized [29,30]. Therefore, it is essential to convert low-frequency compressed air energy into electrical energy by utilizing energy-harvesting devices. 

In addition, the U-shaped tube is a common structure in both industrial and daily life. It has the characteristics of a compact structure, relatively small space occupation, and ease of integration and encapsulation. Under the action of the pneumatic system, the liquid in the U-shaped tube oscillates. Based on the triboelectric effect of the liquid–solid interface, the compressed air energy of the pneumatic system can be harvested. [31,32]. 

**Figure 1:**
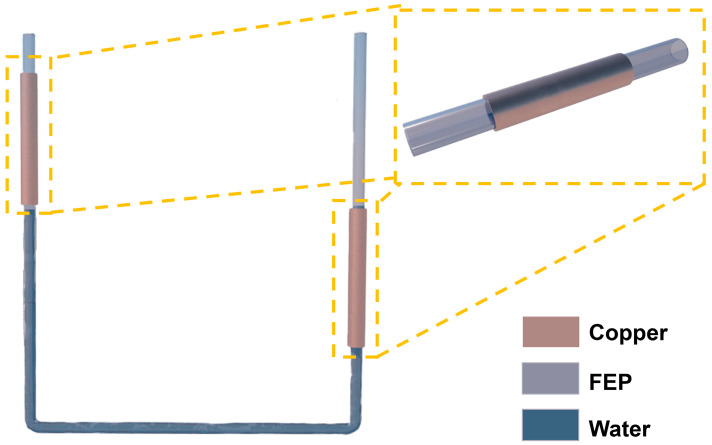
Schematic diagram of the UFC-TENG.

The UFC-TENG device designed in this work is shown in Figure 1, which consists of a U-shaped tube and two copper foil electrodes. The longitudinal height of the U-shaped tube is 25 cm, and the horizontal length is 20 cm. The U-shaped tube is equipped with symmetrical and staggered copper foil electrodes at both ends. The UFC-TENG is a sliding mode freestanding triboelectric nanogenerator [33] based on the triboelectric effect [34]. With the relative movement of contact separation between the frictional layers of the liquid inside the tube and the tube wall, electrons continuously transfer between the electrodes. So far, the mechanism of charge transfer during liquid–solid contact electrification is not clear, but the fact that FEP contacting with water leads to charge transfer at the electrode is not in doubt [35]. 

**Figure 2:**
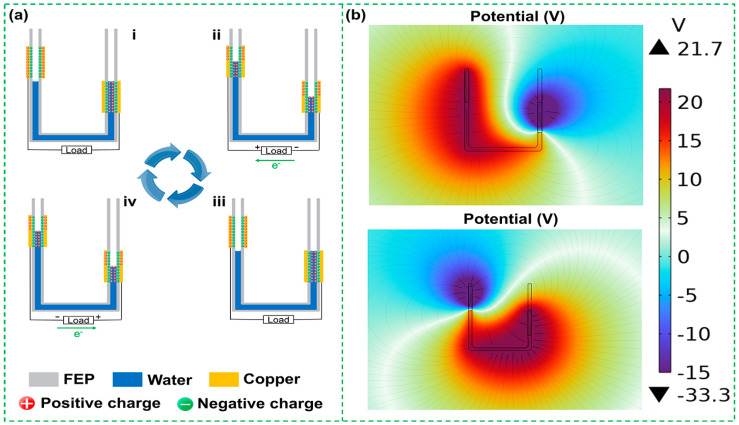
Working mechanism of the UFC-TENG. (**a**) Schematic charge distribution and current direction of the UFC-TENG based on a ring structure with different statuses. (**b**) Between FEP and liquid, potential distribution is simulated by COMSOL 6.0 software in different positions.

The triboelectrification process during the operation of the UFC-TENG is illustrated in Figure 2a. Based on the fundamental principles of hydrostatics, when the pressure changes at one end of the U-shaped tube, the liquid inside the tube will move along the direction of lower pressure. The water contacts and separates from the frictional layer of the tube wall, generating charges that flow out from the electrodes and move along the external circuit. When water flows over the surface of FEP and is about to separate from it, the FEP becomes electronegative while the water becomes electropositive. At this time, water is near the top of the positive charge in the triboelectric sequence (that is, easy-to-carry positive charges) due to it being a strongly polar molecule [36]. FEP has a strong affinity for negative charges, and it also has low surface energy. When water is in contact with FEP, there is a strong overlap of the electron cloud at the interfaces. When static pressure on one end of the U-shaped tube decreases (from i to ii), the positively charged water moves towards the end where the pressure decreases and reaches the highest edge of the copper foil electrode. When the liquid–solid interface at this end is not separated, there is an excess of positive charges remaining in water and negative charges on the surface of FEP, with equal and opposite electric charges attracting each other, forming an electrical double layer (EDL). Simultaneously, the liquid and the surface of FEP at the other end begin to separate. According to the electroneutrality law, FEP that has lost positive charges will induce positive charges to maintain the charge balance. The electric potential of electrodes drives electrons to move directionally along the external circuit, thereby generating electricity. When the static pressure on the liquid at the end, where the static pressure of the U-shaped tube decreases (from iii to iv), no longer changes, the water moves in the opposite direction under the action of gravity. At this point, the process of charge generation is the same as above, but the direction of the current is opposite. When the liquid returns to its original state, the liquid inside the U-shaped tube undergoes an alternating upward and downward movement process. Meanwhile, the UFC-TENG undergoes a complete cycle of charge generation. Based on the above process, the UFC-TENG converts the pressure energy of compressed air in the pneumatic system into electric energy under the action of liquid–solid contact electrification.

COMSOL Multiphysics 6.0 software is used for finite element analysis, displaying the simulated potential distribution on a color scale. When water reaches the different positions of the electrodes on both sides, there is a potential distribution of the UFC-TENG between the liquid and FEP, as shown in Figure 2b. This indicates that the potential difference between FEP and water is large.

### 3.2. Optimization of the UFC-TENG

#### 3.2.1. Influence of Tube Materials and Water Quality

**Figure 3:**
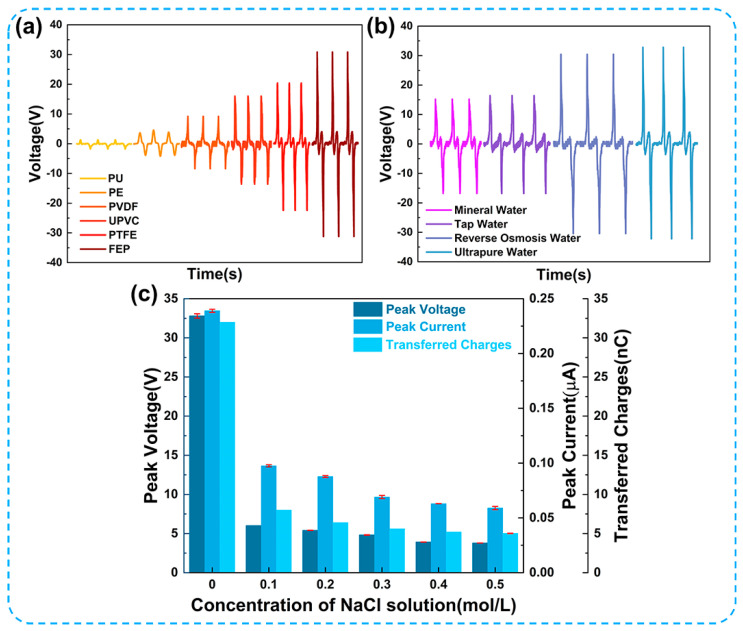
Electrical output of the UFC-TENG of different triboelectric materials. Output voltage of UFC-TENG under (**a**) different tube materials and (**b**) different water qualities. (**c**) Peak voltage, peak current, and transferred charges under different concentrations of NaCl solution.

The formation of the EDL is attributed to the transfer of electrons and ions at the solid–liquid interface. Different materials for triboelectric nanogenerators possess distinctive characteristics in terms of electron affinity, surface energy, permittivity, and hydrophobicity [37]. Wang et al. [38] have confirmed that the contact electrification between liquids and polymers is related to electron-withdrawing groups in polymers. Figure 3a,b show that different tube materials and water quality significantly affect the output performance of the UFC-TENG. Figure 3a demonstrates that the UFC-TENG using FEP as a triboelectric material exhibits the best electrical output, followed by PTFE, UPVC, and PVDF, while PE and PU have the worst output performance. This is due to the gradual increase in the fluorine functional groups contained in PU, PE, PVDF, UPVC, PTFE, and FEP. Among them, the trifluoromethyl group in FEP has the strongest ability to absorb electrons and can provide the highest charge density. In addition, the excellent surface hydrophobicity of FEP is also an important factor because it has the best output performance. The contact angle between FEP and water is shown in Figure S4. The output performances of TENGs using mineral water and tap water are significantly decreased, except for ultrapure water, as shown in Figure 3b. This may be due to the result of a large number of free ion groups in the liquid that shield the EDL at the liquid–solid interface. Through several experiments on changing the concentration of NaCl solution within the U-shaped tube, it was observed that ion groups have a certain influence on the output performance of the UFC-TENG. The experimental result is illustrated in Figure 3c. It can be seen that there is a negative correlation between the concentration of NaCl solution and the electrical output of the UFC-TENG. As the concentration of NaCl solution increases from 0 to 0.5 mol/L, the peak voltage (*V_pk_*), peak current (*I_pk_*), and transferred charges (*Q_tr_*) decrease from 32 V to 3.8 V, from 0.23 μA to 0.06 μA, and from 32 nC to 5 nC, respectively. Previous studies [39,40] have also confirmed that increasing the concentration of ions can inhibit the electron transfer at the liquid–solid interface, which proves that the performance of L-S TENGs can be affected by saline solution.

#### 3.2.2. Influence of Tube Structure Parameters

**Figure 4:**
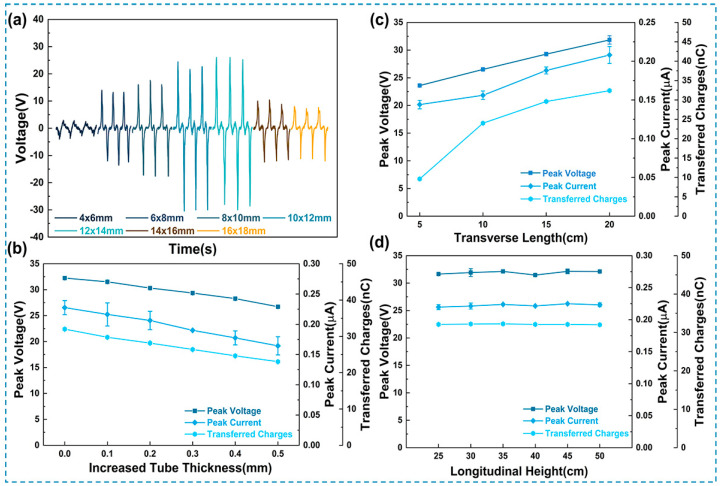
Electrical output of the UFC-TENG under different tube structures. (**a**) Output voltage of the UFC-TENG with different FEP inner and outer diameters. (**b**) Peak voltage, peak current, and transferred charges for the UFC-TENG tubes measured at various FEP thicknesses. (**c**) Peak voltage, peak current, and transferred charges with increasing transverse length of UFC-TENG. (**d**) Peak voltage, peak current, and transferred charges with increasing longitudinal height of UFC-TENG.

In addition to the significant impact of triboelectric materials on the performance of the UFC-TENG, it was also found that the structural parameters of the tube have a great influence on the performance of the UFC-TENG. As shown in Figure 4a, the open-circuit voltage of the UFC-TENG has different tube diameters and tube thicknesses. Under the condition of ensuring that the tube thickness remains 1 mm, the electrical output of the UFC-TENG increases first and then decreases as there is an increase in the tube diameters (including the inner diameter and outer diameter). This indicates that there is an optimal value for the tube diameter of UFC-TENG. The reason for this is that the contact effectiveness between the tube wall and the liquid increases when the tube diameter increases. When the inner diameters of the U-shaped tube are 10 mm and 12 mm, the open-circuit voltages of the UFC-TENG are both high. However, with the further increase in the tube diameters, the pressure difference at both ends of the U-shaped tube may not ensure that the electrodes are completely covered when the liquid is sliding, resulting in a relatively poor output performance. In addition, by comparing the short-circuit current and the transferred charges (as shown in Figure S2), it was found that the UFC-TENG with inner and outer diameters of 12 mm and 14 mm shows the best output performance. Therefore, in the following experiments, the inner and outer diameters of the UFC-TENG in the subsequent experiments of this work are 12 mm and 14 mm, unless otherwise stated.

As shown in Figure 4b, it can be observed that increasing the tube thickness leads to a decrease in the peak voltage, keeping the inner diameter constant. This is because the contact between the liquid and the tube wall is achieved through electrostatic force, and the magnitude of the electrostatic force is inversely proportional to the distance (tube thickness) between the triboelectric charges and the measured position [41]. Therefore, when the thickness of the FEP tube increases, the electrostatic force acting on it by the liquid decreases, resulting in a decrease in the number of charges generated by electrostatic induction. On the contrary, when the thickness of the FEP tube decreases, the electrostatic force acting on it by the liquid increases, resulting in an increase in the number of induced charges generated by electrostatic induction.

To further explain the influence of tube structure parameters on the electrical output of the UFC-TENG in detail, the effects of transverse length and longitudinal height on the electrical output of the UFC-TENG were also studied. The electrical performance of the UFC-TENG was tested on the premise that the pressure difference between both ends of the U-tube remained constant. It can be seen from Figure 4c that there is a variation in *V_pk_*, *I_pk_*, and *Q_tr_* with an increase in the transverse length. With the increase in the transverse length, the total length of the liquid inside the U-shaped tube increases. This causes the alternating frequency of the liquid to decrease, as shown in Equation (1) [42]:

(1) $$f=\frac{1}{2\pi }\sqrt{\frac{2g}{L}}$$

where *f* is the alternating frequency of the liquid inside the U-shaped tube, *L* is the total length of the liquid inside the U-shaped tube, and *g* is the acceleration of gravity.

In Figure 4c, the length of the liquid inside the UFC-TENG when the transverse length of the tube is 5 cm is shorter than that when the transverse length of the tube is 10 cm. The increased frequency of the liquid results in a short-distance displacement. The liquid cannot completely cover the copper foil electrode (i.e., the effective contact area for triboelectrification). Therefore, the UFC-TENG has a few transferred charges, limited by both the effective contact area for triboelectrification and the alternating frequency of the liquid. However, when the transverse length of the tube increases to 10 cm, the length of the liquid inside the tube also increases. At this time, the alternating frequency of the liquid decreases, the distance of the liquid alternation increases, and a longer time interval allows the liquid to completely cover the copper foil electrode, resulting in an increase in transferred charges. In this case, the effective contact area for triboelectrification increases and the alternating frequency of the liquid decreases, resulting in a greater value of transferred charges. After further increasing the transverse length of the tube, this only changes the alternating frequency of the liquid, while the effective contact area for triboelectrification remains unchanged. The increase in transferred charges is less.

As can be seen from Figure 4d, the variations in the longitudinal height of the U-shaped tube have little effect on the electrical output of the UFC-TENG. This is because variations in the longitudinal height of the U-shaped tube would not affect the contact effectiveness between the liquid and the tube wall. As long as the pressure difference at both ends of the U-shaped tube remains unchanged, the output of the UFC-TENG will hardly change with the longitudinal height of the U-tube.

#### 3.2.3. Influence of Liquid Flow Characteristics

**Figure 5:**
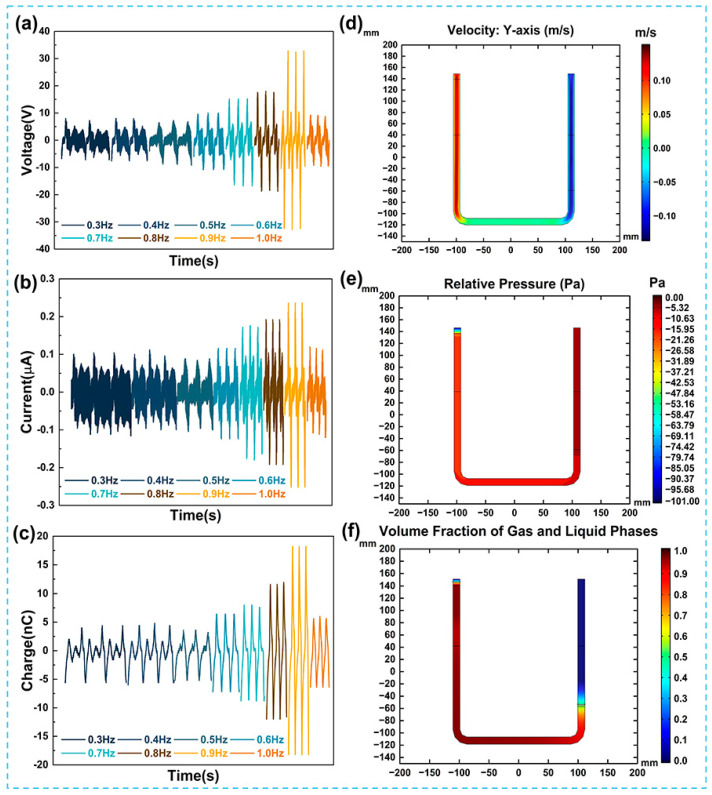
Electrical output of the UFC-TENG with the influence of liquid flow characteristics. (**a**) Voltage, (**b**) current, and (**c**) charges of the UFC-TENG under different operating frequencies. (**d**) Velocity (along the y-axis), (**e**) pressure (relative pressure), and (**f**) volume fraction distribution of gas–liquid at the left highest point of UFC-TENG.

In the aforementioned work, it was preliminarily indicated that the liquid flow characteristics can affect the contact electrification liquid–solid interface and the electrical output of the UFC-TENG. Figure 5a–c, respectively, show the open-circuit voltage, the short-circuit current, and the transferred charges of the UFC-TENG under different liquid alternating frequencies. When the alternating frequency of the liquid is lower than or equal to 0.9 Hz, the electrical output of the UFC-TENG improves with the acceleration of alternating frequency of the liquid. However, the situation at *f* = 0.5 Hz is special. At this time, the effect of the inertial force generated by the acceleration of gravity makes the charges of the UFC-TENG less those that at other liquid alternating frequencies. When the liquid falls back down under the combined effect of gravity and inertial force, the liquid level is lower than in its original state. Therefore, the effective contact area decreases at the liquid–solid interface when the liquid moves upwards due to the entrainment. The reduction in the effective friction area and the weakening of the alternating frequency of the liquid jointly lead to a decline in the electrical output of the UFC-TENG. In other cases where the alternating frequency of the liquid is below 0.9 Hz, the factors affecting the output performance of the UFC-TENG are only related to the alternating frequency of the liquid. When *f* = 0.9 Hz, the *V_pk_* of the UFC-TENG is the highest, and the maximum value is 33 V. As there is an increase in the alternating frequency of the liquid, the effective friction area between the liquid and tube wall decreases, which leads to the deterioration of the output performance of the UFC-TENG. To obtain the best electrical output of the UFC-TENG, a liquid alternating frequency of 0.9 Hz is selected as the operating frequency of the TENG.

In COMSOL Multiphysics 6.0 software, the two-phase flow physical field is used to describe the fluid dynamics characteristics and define the fluid types of gas and liquid. As shown in Figure 5d–f, the liquid velocity along the y-axis direction, pressure, and gas–liquid volume fraction were all obtained at the same time (taking the liquid moving to the highest point on the left as an example). It can be seen in Figure 5d that the closer the liquid at the right end is to the lowest end of the copper electrode, the higher the liquid velocity is along the y-axis direction. Under the condition that the alternating frequency of the liquid remains unchanged, the higher the liquid velocity, the faster the formation rate of the EDL, and the more transferred charges there are, at the liquid–solid interface in unit time. At this time, the short-circuit current of the UFC-TENG also increases.

#### 3.2.4. Stability Testing

The stability testing of the device is a necessary step to verify whether the L-S TENG can be used in practical applications for a long time. It is well known that the non-robustness of contact electrification at the liquid–solid interface leads to charge loss due to electrostatic induction (such as the interference of environmental factors, e.g., air humidity), which seriously affects the sensitivity and reliability of TENGs as energy-harvesting devices.

**Figure 6:**
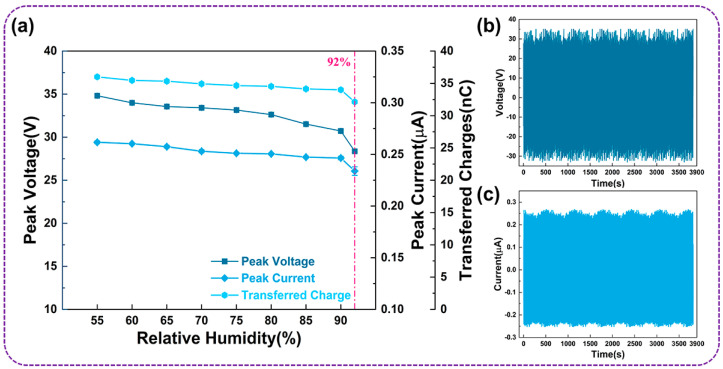
The stability testing of the UFC-TENG. (**a**) The influence of relative humidity on the electrical output of the UFC-TENG. (**b**) Voltage and (**c**) current of the UFC-TENG are continuously monitored for over an hour of operation.

Figure 6a illustrates the influence of ambient relative humidity on the electrical output performance of the UFC-TENG. When the relative humidity is between 55% and 90%, the peak voltage, peak current, and transferred charges gradually decrease, but the reduction is not significant. When the relative humidity exceeds 90% (the maximum humidity in this work is increased to 92%), the peak voltage, peak current, and transferred charges significantly decrease. This is due to the high humidity causing water vapor to adhere to the inner wall of the U-shaped tube. A large amount of water molecules accumulate on the surface of the frictional layer and form a layer of water film. The water film depletes the triboelectric charge on the surface of the frictional layer, resulting in a decrease in the output performance of the UFC-TENG. 

Furthermore, after continuous operation for over an hour at a frequency of 0.9 Hz, the open-circuit voltage and short-circuit current of the UFC-TENG hardly change, indicating its reliable stability (as shown in Figure 6b,c). On the one hand, the special shape of the U-shaped tube effectively guides the liquid, keeping it flowing smoothly inside the tube. On the other hand, the U-shaped tube shows good structural strength and can withstand high pressure, making it suitable for high-pressure conditions. Moreover, the curved shape of the U-shaped tube generates a certain buffering effect when the liquid passes through it. In the case of sudden pressure changes, the buffering effect of the U-shaped tube can reduce the pressure changes in the liquid and partly prevent bubbles from being generated during the alteration of the liquid. Therefore, the liquid inside the U-shaped tube is easier to control, which is beneficial for the research of the relevant properties of the liquid. In a word, the liquid–solid triboelectric nanogenerator based on a U-shaped tube can demonstrate superior environmental adaptability.

### 3.3. Applications of the UFC-TENG

In addition to studying the fundamental performance of the UFC-TENG, it is also important to study its practical application. The *V_pk_* and *I_pk_* of the UFC-TENG under different external load resistances were tested in this work. The output power can be obtained from Equation (2) as follows:

(2) $$P=\frac{U^{2}}{R_{L}}$$

where *P* represents the output power, *U* represents the peak voltage, and *R_L_* represents the external load resistances of the UFC-TENG.

**Figure 7:**
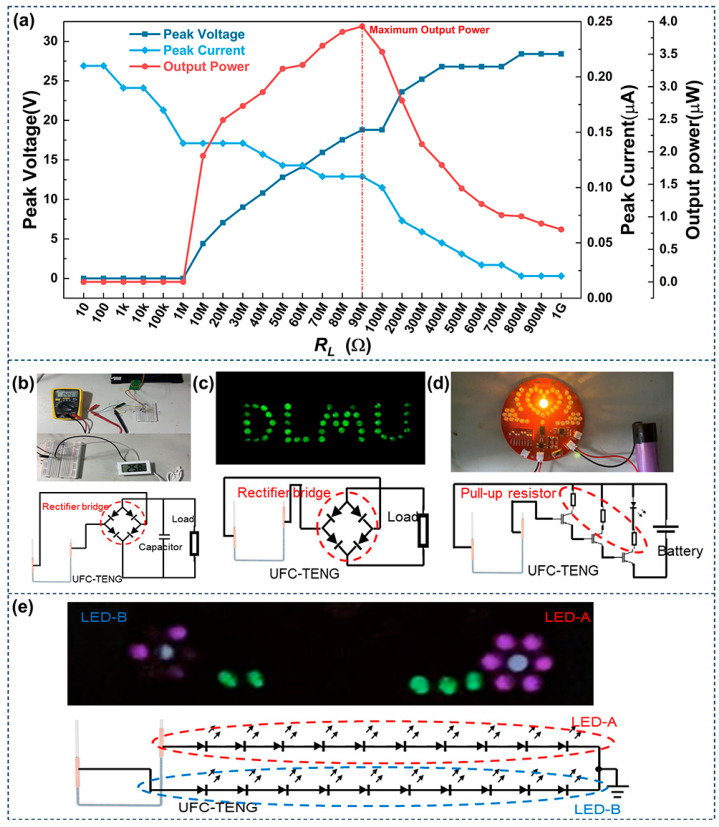
Applications of the UFC-TENG. (**a**) Peak voltage, peak current, and output power of UFC-TENG under different loads. The corresponding circuit schematic diagram where UFC-TENG is used as (**b**) a power supply to (**c**) light up LEDs and (**d**) as a signal collector, which is amplified by an amplifier. (**e**) UFC-TENG is used as a self-powered sensor in the corresponding circuit schematic diagram.

As shown in Figure 7a, there are variations in the peak voltage, peak current, and output power with different external load resistances. It can be observed that the peak voltage increases with the increase in external load resistances, while the peak current decreases with the increase in external load resistances. The output power of the TENG is optimal when the external load resistance is 90 MΩ, reaching a maximum value of 3.93 μW. According to the maximum power transfer theorem, when the load resistance of the external circuit is equal to the internal resistance of the power supply, the external load obtains maximum power. Therefore, the resistance of the UFC-TENG is 90 MΩ in this work. As illustrated in Figure S3, the UFC-TENG can produce electricity and store it in a capacitor. Furthermore, the UFC-TENG can charge a 1 μF capacitor to 1.25 V or higher within 20 s. It can also be found that the charging speed of the UFC-TENG will obviously decrease as the capacitance increases (Figure S3). The above results demonstrate that the UFC-TENG can convert the unutilized compressed air energy in pneumatic systems into electric energy. Here are several demonstrations to display various applications of the UFC-TENG:

(1) The UFC-TENG can store electric energy in capacitors and use it to drive small electronic devices such as sensors. As shown in Figure 7b, the alternating current of the UFC-TENG is rectified by a full wave rectifier circuit and charged by a 100 μF capacitor. Subsequently, the connection between the UFC-TENG and the capacitor is disconnected, and the capacitor is connected to a temperature and humidity sensor. The capacitor can drive a simple temperature and humidity sensor.
(2) The UFC-TENG can be used as a DC power supply to light up LEDs. LED lights are very common in our daily lives, serving various purposes such as creating atmospheres, decorating storefronts, and providing illumination. The UFC-TENG designed in this work can easily light up at least 40 LEDs after being rectified by a full wave rectifier circuit. The corresponding demonstration and circuit diagram are shown in Figure 7c and Video S1.
(3) The UFC-TENG can be used as a signal collector and realize signal analysis and processing by connecting with other devices. Low-frequency signals can penetrate obstacles during the transmission process, demonstrating strong penetration ability and long transmission distances. The UFC-TENG can use an amplifier composed of transistors to acquire low-frequency signals. The corresponding circuit schematic diagram is illustrated in Figure 7d, and a detailed video demonstration is shown in Video S2. It can be seen from the demonstration that the UFC-TENG can transform the mechanical signals of liquid motion into weak electrical signals and be amplified through amplification circuits. As the alternating frequency of the liquid changes, the brightness flashing times of the small bulb are different. When the liquid is in its initial state and stops moving, the small bulb no longer lights up.
(4) The UFC-TENG can be used as a self-powered sensor. With the rapid development of the Internet of Things, a huge number of sensors are distributed in every corner of the world. It is almost impossible to provide power for this amazing number of sensors with traditional batteries. Therefore, many scholars have dedicated themselves to the research of self-powered sensors. By connecting the UFC-TENG to the circuit shown in Figure 7e, when the liquid inside the UFC-TENG moves towards the right end, the current flows in the direction of the grounding end. At this time, the LED-A circuit is switched on, and the LEDs on this circuit light up. On the contrary, when the liquid inside the UFC-TENG moves towards the left end, the current flows in the direction of outflow from the ground end. At this time, the LED-B circuit is switched on, and the LEDs on this circuit light up. When the liquid alternately rises inside the UFC-TENG, the LEDs on the LED-A and LED-B circuits light up alternately. Therefore, observing the changes in the quantity and brightness of small light bulbs of LEDs can allow for the operation status of the equipment integrated with the UFC-TENG to be monitored.

## 4. Conclusions

In summary, the UFC-TENG designed in this work can effectively harvest compressed air energy discharged to the atmosphere from pneumatic systems. The total length of the UFC-TENG is 70 cm, and the tube thickness is 1mm, while the inner diameter of the U-shaped tube is 12 cm. FEP and ultrapure water were selected as the triboelectric materials. The experimental results demonstrate that the characteristics of the triboelectric materials and liquid flow of the U-shaped tube significantly affect the electrical output of the UFC-TENG. When RL is 90 MΩ, the maximum output power is about 3.93 μW. The optimized UFC-TENG can be used as a capacitive storage device to power the temperature and humidity sensors, light up LED lights, and serve as a signal acquisition device. The UFC-TENG can provide an effective strategy for energy transformation in pneumatic systems due to its simple manufacturing, good abrasion resistance, and ease of integration. This work expands the application fields of TENGs and demonstrates the promising potential of L-S TENGs in the fields of energy harvesting and sustainable development.

## Data Availability

The data are available upon reasonable request from the corresponding author.

## Supplementary Materials

The following supporting information can be downloaded at: https://www.mdpi.com/article/10.3390/mi14112057/s1, Figure S1: Schematic diagram of the UFC-TENG testing system. (a) Schematic diagram of the basic structure of the two-position, five-way pneumatic control valve. (b) Schematic diagram of the basic structure of the ejector. Figure S2: Current (a) and charges (b) of the UFC-TENG with different inner and outer diameters. Figure S3: Charging curves of different capacitors. Figure S4: Contact angle between FEP and water. Video S1: Demonstrate that the UFC-TENG is used as a DC power supply to light up LEDs after a full wave rectifier circuit; Video S2: Demonstrate that the UFC-TENG is used as a signal collector amplified by triodes and converted into electrical signals.
