# Supplementary material for: U-Shaped Tube Based Liquid–Solid Triboelectric Nanogenerator for Harvesting Unutilized Compressed Air Energy
Source: Micromachines (Basel). 2023 Nov 2;14(11):2057. doi: 10.3390/mi14112057 (PMC10673232; doi:10.3390/mi14112057)
Supplement: Supplementary file 1 [file micromachines-14-02057-s001.zip › micromachines-2660948-supplementary.pdf]

Supporting Information

U-Shaped Tube Based Liquid–Solid Triboelectric Nanogenerator for Harvesting Unutilized Compressed Air Energy

Xuhang Cai, Zhijian Liu *, Jingming Dong *, Haoji Li, Jiamu Han, Jiaming Huang and Haotian Chen

Marine Engineering College, Dalian Maritime University, Dalian 116026, China; dmucxh@dlmu.edu.cn (X.C.); 0741lhj@dlmu.edu.cn (H.L.); han0814@dlmu.edu.cn (J.H.); jiaming_huang@dlmu.edu.cn (J.H.); hotachen@dlmu.edu.cn (H.C.)

* Correspondence: liuzhijian@dlmu.edu.cn (Z.L.); dmudjm@dlmu.edu.cn (J.D.)

Supporting files

Supporting Figures: **Figure S1-S4**

Supporting Video: **Video S1-S2**

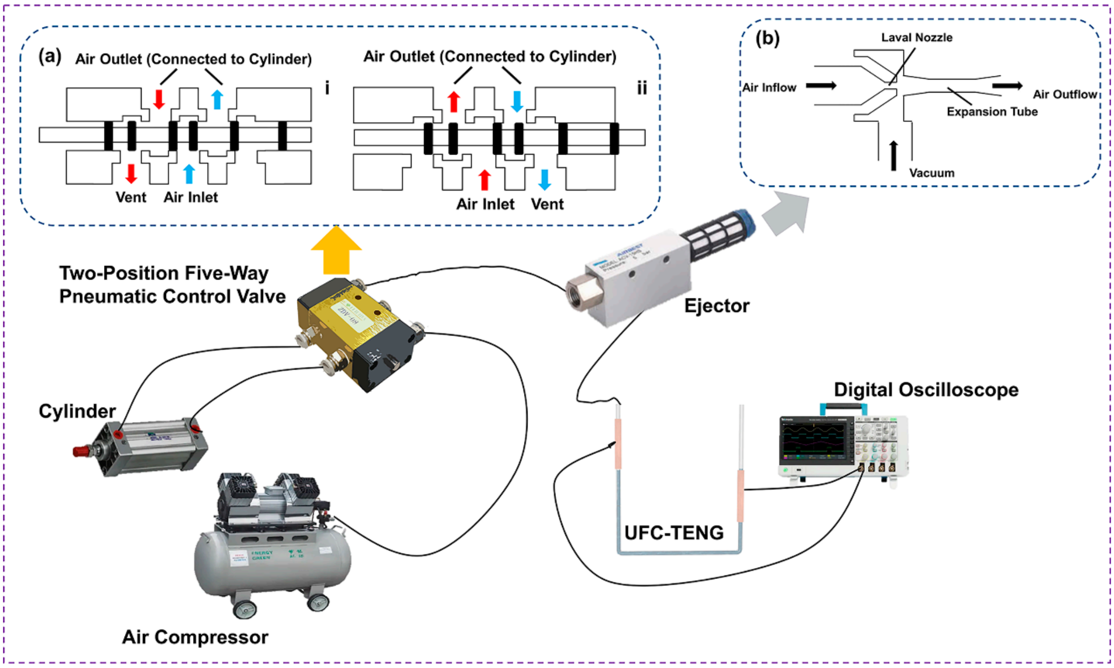

Figure S1 Schematic diagram of the UFC-TENG testing system. (a) Schematic diagram of the two-position five-way pneumatic control valve. (b)Schematic diagram of the ejector.

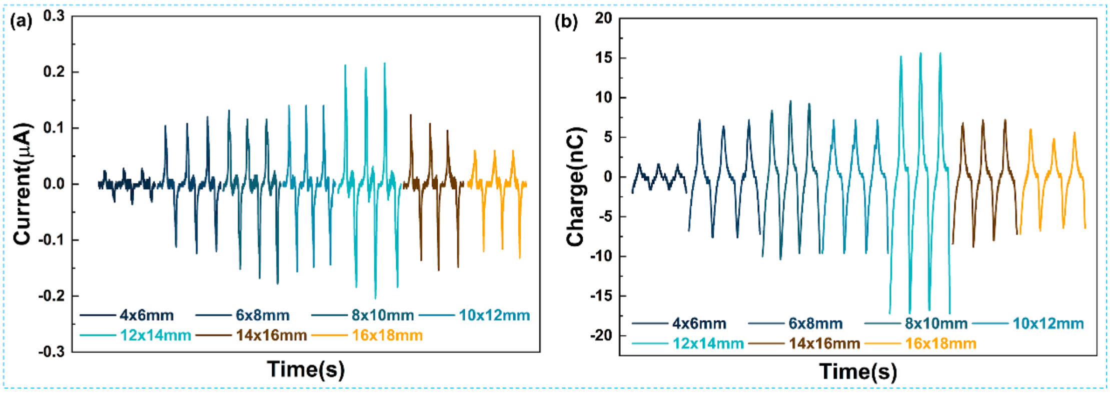

Figure S2 Current (a) and charges (b) of the UFC-TENG with different inner and outer diameters.

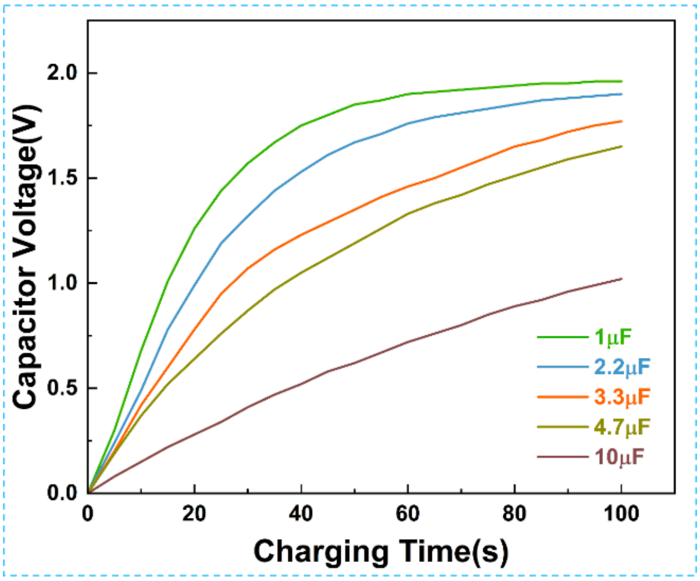

Figure S3 Charging curves of different capacitors.

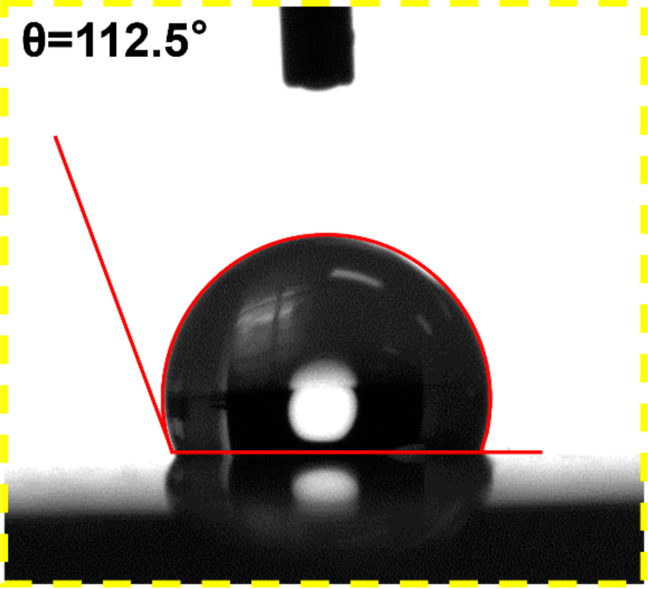

Figure S4 Contact angle between FEP and water.

Supporting Video

Video S1 Demonstrate that the UFC-TENG is used as a DC power supply to light up LEDs after full wave rectifier circuit.

Video S2 Demonstrate that the UFC-TENG is used as a signal collector, which is amplified by triodes and converted into electrical signals.
